# Case Report: Transanal extraction combined with wound management for a broken glass foreign body and associated rectal injuries

**DOI:** 10.3389/fsurg.2026.1803580

**Published:** 2026-05-07

**Authors:** Long Chen, Yuhua Zhang, Wenyan Zhou, Xiaohui Ji, Yongchao Lu, Zhongbao Niu

**Affiliations:** 1 The First Clinical Medical College, Shandong University of Traditional Chinese Medicine, Jinan, Shandong, China; 2 Department of Traditional Chinese Medicine (Proctology Division), Shandong Provincial Hospital Affiliated to Shandong First Medical University, Jinan, Shandong, China

**Keywords:** case report, debridement, ligation, rectal foreign body, rectal lacerations, transanal removal

## Abstract

**Background:**

Rectal foreign bodies (RFBs) have become a relatively common yet often underestimated clinical entity. Fragile and sharp objects like glass pose a perplexing challenge, as unsafe removal may lead to complications or even life-threatening injuries. This report details the management of a complex case involving a broken glass rectal foreign body with associated rectal injuries.

**Case presentation:**

A 55-year-old male was brought to the emergency department complaining of rectal fullness and bleeding after inserting a glass cup. Plain abdominal X-ray film showed an irregular dense shadow approximately 7 cm in length within the pelvis, with no evidence suggesting bowel perforation. A rectal digital examination (DRE) revealed the presence of an intrarectal foreign body with suspected rectal mucosal injury. The foreign body was successfully removed transanally under spinal anesthesia. Assessment revealed rectal lacerations without active bleeding. To mitigate the risk of entrapping minute glass fragments, the wounds were debrided and left open for secondary intention healing. Furthermore, we selectively ligated the congested and redundant mucosal folds adjacent to the wounds to promote healing. The postoperative course was uneventful. Flexible proctosigmoidoscopy at two-month follow-up demonstrated complete mucosal healing without complications.

**Conclusions:**

The management of broken glass RFBs with associated rectal injuries requires a systematic approach involving precise diagnosis, cautious intervention, and attentive postoperative care. This case describes a distinct management strategy for such injuries and will provide a reference for clinicians facing similar clinical scenarios.

## Introduction

Rectal foreign bodies (RFBs), first reported in the 16th century (1), have become a relatively common yet often underestimated clinical entity (2). Retention of RFBs has diverse etiologies, which can be broadly categorized into sexual purposes, accidents (including ingestion), assistance with defecation, and criminal assaults (3). Owing to shame and embarrassment, patients frequently attempt self-removal and delay seeking hospital care (4), leading to underreporting and a systematic underestimation of the true incidence. Reported studies indicate a marked male predominance in this condition. The most frequent motive is sexual pleasure (5), and the most commonly encountered foreign bodies are sex toys and glass objects (6). Additionally, this clinical scenario becomes particularly challenging in cases where psychiatric illness or intellectual disabilities exist (3, 7).

Most blunt foreign bodies can be safely removed via a transanal approach. In contrast, glass objects pose a unique clinical challenge due to their friability and sharp nature, as accidental breakage can result in pain, rectal bleeding, fragment retention, and even serious rectal injuries (8). The management should be guided by established trauma principles. The Eastern Association for the Surgery of Trauma (EAST), for instance, has published practice management guidelines for penetrating extraperitoneal rectal injuries (9), which help address the key questions a surgeon faces: how to safely remove the object, whether fecal diversion or presacral drainage is necessary, and whether primary repair of associated lacerations is feasible (9). This report describes a case of a broken glass foreign body with associated rectal injuries, detailing a treatment strategy of transanal removal, wound debridement, and mucosal ligation resulting in an uncomplicated recovery.

## Case presentation

A 55-year-old male was brought to the emergency department with a one-day history of rectal fullness and bleeding. He admitted having self-inserted a glass cup base-first into his rectum the previous day for sexual stimulation, which he was unable to retrieve. Several unsuccessful self-removal attempts resulted in breakage of the cup and minor bleeding, prompting his visit.

**Figure 1:**
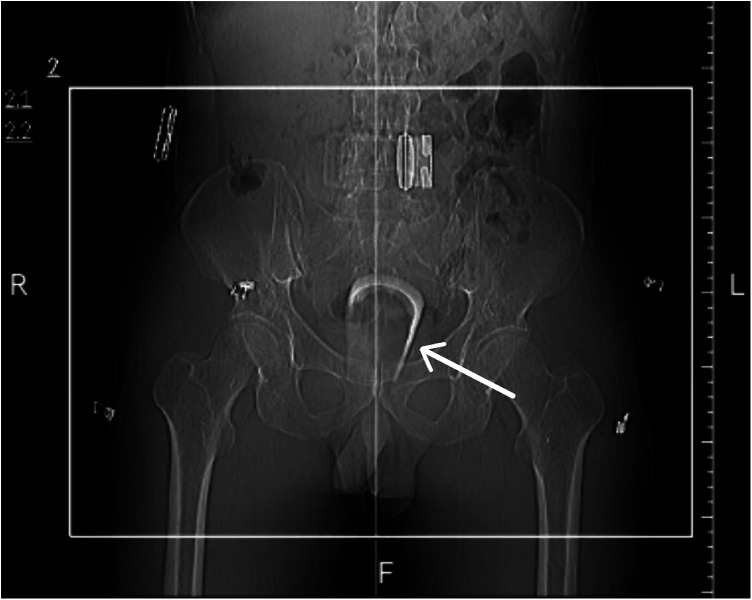
Plain abdominal X-ray film revealed an irregular dense shadow approximately 7 cm in length within the pelvis (white arrow).

The patient had no remarkable past medical history aside from a remote history of left heel surgery. On admission, he was oriented, composed, speaking rationally, and did not demonstrate any signs or symptoms of psychosis. He reported no history of smoking, alcohol consumption, or illicit drug use. His vitals and laboratory findings were within normal limits. Routine preoperative screening for infectious disease (HBV, HCV, HIV, syphilis) was negative. Abdominal examination revealed no abnormalities. However, a plain abdominal X-ray film revealed an irregular dense shadow approximately 7 cm in length within the pelvis (Figure 1), with no evidence of extraluminal air or free fluid to suggest bowel perforation. On digital rectal examination (DRE), a hard, hollow, and immobile object was palpated approximately 1.5 cm from the dentate line, embedded obliquely within the distal rectum and partially broken. Bright red blood was noted on the glove upon withdrawal. The diagnosis of a rectal foreign body, specifically a base-up positioned broken glass cup, was established based on the patient history and imaging findings. Rectal bleeding raised suspicion for associated rectal injuries.

**Figure 2:**
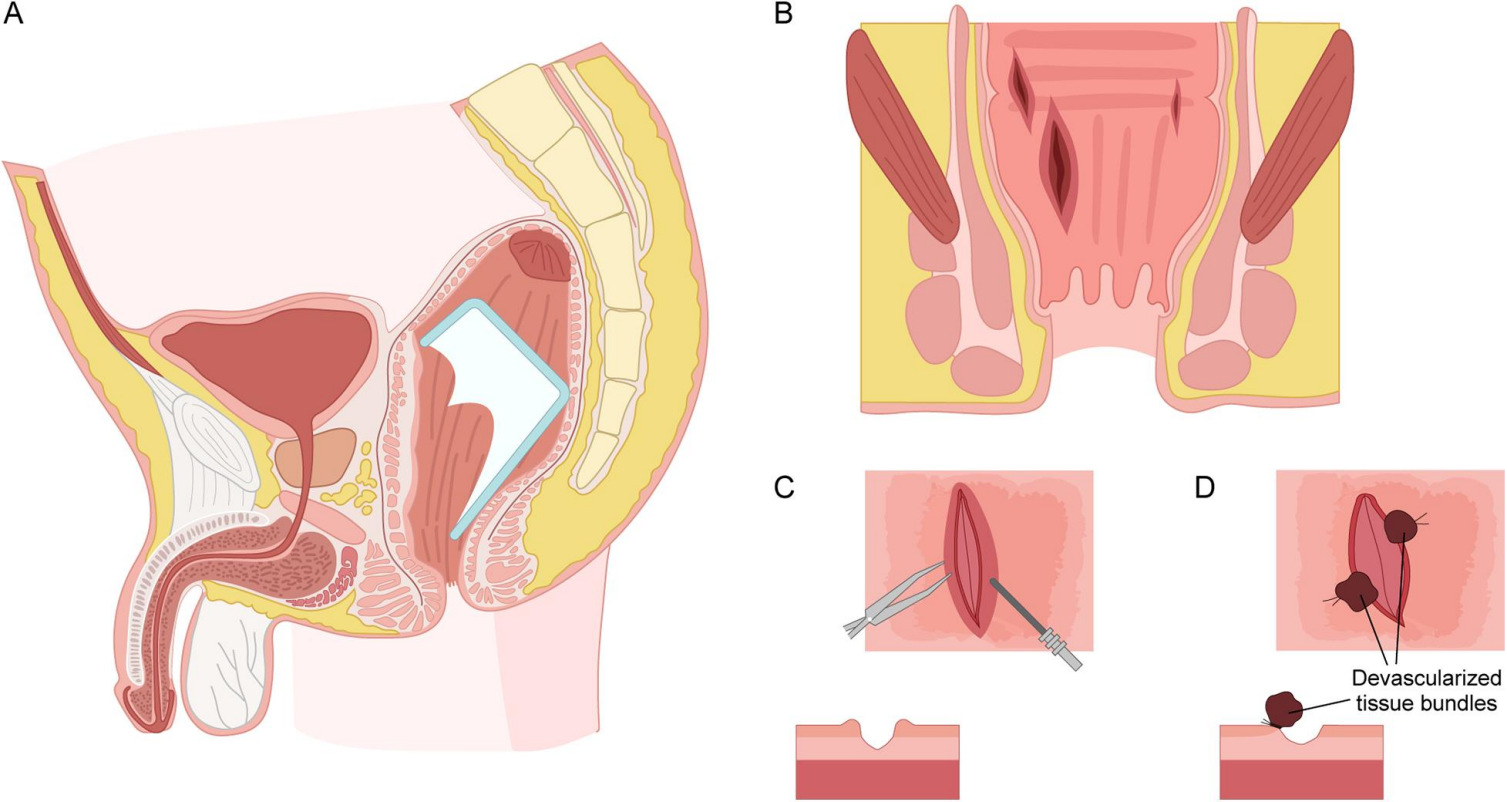
Schematic illustration of the impaction of the rectal foreign body and the management of associated rectal injury. **(A)** The foreign body (a broken glass cup with a missing segment) impacted in the distal rectum. **(B)** Lacerations above the dentate line with the adjacent tissue congestion and edema. **(C)** Debrided wound with a V-shaped cross-section. **(D)** Ligation of the congested and redundant mucosal folds near the wounds.

**Figure 3:**
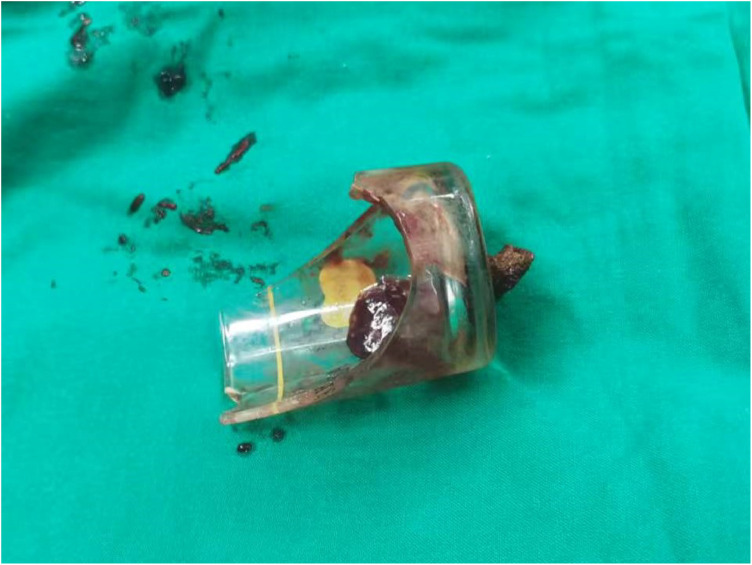
The removed foreign body.

The patient underwent transanal removal of the foreign body in the operating room. Preoperative management included fasting, intravenous nutritional support, and broad-spectrum antibiotic prophylaxis (cefuroxime). Under spinal anesthesia in the lithotomy position, the perianal skin and anal canal were draped after careful disinfection. Adhesive surgical films were used to retract the bilateral buttocks for optimal exposure. Using thyroid retractors, a broken glass cup with a missing segment was visualized in the distal rectum (Figure 2A). The fractured edge had created a suction effect on the adjacent mucosa, which appeared markedly congested and edematous. First, we gently dissected the interface between the sharp glass rim and the mucosa with digital manipulation. A moistened sterile gauze was then interposed to shield the mucosa from the sharp edges. Subsequently, the remaining intact portion of the glass cup was gently grasped with an oval forceps. Controlled traction was applied and en-bloc removal was achieved (Figure 3). Following removal, the rectal lumen was copiously irrigated with normal saline and thoroughly inspected to exclude retained fragments.

The rectal injury and mucosal integrity were then assessed. Above the dentate line, localized areas of the mucosa showed significant congestion and edema with several spindle-shaped lacerations, which were pronounced in the right posterior rectal wall (Figure 2B). Systematic assessment revealed that the injuries were confined to the mucosal and submucosal layers, consistent with a grade I rectal injury (partial-thickness laceration) according to the American Association for the Surgery of Trauma (AAST) organ injury scale. No active bleeding was noted. Meticulous debridement was performed with cutting electrocautery to trim all devitalized mucosal edges back to viable tissue and fashion the wounds into a V-shaped configuration (Figure 2C). To avoid entrapping any potential minute glass fragments, the wounds were kept open rather than undergoing primary suture closure. Pinched with the tissue forceps, the adjacent edematous mucosal folds and their vascular bases were selectively ligated with non-absorbable sutures, creating well-demarcated, devascularized tissue bundles (Figure 2D), which were expected to slough off spontaneously. After achieving complete hemostasis, a loose Vaseline gauze dressing was placed in the rectum. The perianal area was covered with a sterile dressing secured by a T-bandage.

Postoperative management included daily rectal lavage, wound dressing changes, analgesics, and a 24-hour course of cefuroxime. For edema control, diosmin was administered. Intravenous amino acids and lipid emulsion were given for the first 24 h, followed by gradual diet advancement. Mild laxatives were prescribed to avoid mechanical trauma by hard stools. Given remote residence and limited access to follow-up care, a hospitalization for observation was undertaken. The patient resumed defecation on postoperative day 3 with normal bowel function. He was discharged on postoperative day 5 in stable condition. Pre-discharge anoscopy showed clean, healing wounds. Anal sphincter tone was intact. Although a psychiatric referral was offered, the patient declined. Two months after the procedure, follow-up flexible proctosigmoidoscopy demonstrated complete wound healing with no significant fibrosis or stricture.

## Discussion

Rectal foreign bodies (RFBs) are seen regularly in most large hospitals, although accurate data on their incidence are not accessible as such cases are frequently underreported (10). The literature also shows a male predominance, with reported male-to-female ratios ranging from approximately 6:1 to as high as 37:1 (6, 10, 11). The peak prevalence occurs in the fourth decade of life (4). A wide spectrum of objects has been retrieved, often inserted for sexual gratification. Concerned about the social stigma of public exposure, the patients often delay seeking medical care and try to remove the object alone. Unsuccessful attempts are a major risk factor for complications such as mucosal injury, hemorrhage, and even perforation (12). Accordingly, a high index of suspicion is essential, and a rectal foreign body should be included in the differential diagnosis for patients presenting with unexplained rectal or pelvic pain, fullness, or bleeding.

Glass objects are consistently reported as one of the most common RFBs apart from sex toys, constituting approximately 17.5% of cases (6). They pose a unique clinical challenge during management due to their friability and sharp nature, a risk that is particularly heightened if the glass breaks inside the rectum. Abdominal imaging is warranted to clearly delineate the location and fragmentation of the foreign body, as well as the presence or absence of pneumoperitoneum, hemoperitoneum, or free intra-abdominal fluid. Furthermore, a precise patient history and a thorough physical examination including DRE are indispensable for accurate diagnosis. Abdominal imaging (radiography or computed tomography) should be performed prior to the DRE to provide a safe overview and avoid secondary injury. Under sedation and anesthesia, most RFBs can be removed transanally, either manually or with endoscopic assistance using a colonoscope or sigmoidoscope (13, 14). For instance, Rimsha et al. (15) successfully removed a large, intact glass bottle via the transanal route. Lin et al. (16) retrieved a glass bottle that had migrated deep into the sigmoid colon using gastrolith forceps under colonoscopic guidance. Gupta et al. (17) reported an elderly male with untreated psychiatric illness who accidentally inserted a glass cough syrup bottle into the rectum; the object was successfully removed transanally using obstetric forceps under general anesthesia. In cases where the object is tightly impacted, the suction created by the retained foreign body can make removal difficult. Breaking this air-tight seal is an important step. A catheter with sufficient rigidity (e.g., a suction catheter or urethral catheter) can be advanced between the object and the mucosa to break the seal, which often facilitates smooth extraction. When transanal extraction fails, diagnostic laparotomy or laparoscopy is needed (13, 18). Narjis et al. (19) reported a case in which manual removal of a glass cup from the rectum led to its fragmentation. Given the high risk of further injury, laparotomy for extraction was conducted and a temporary diverting colostomy was created. The current report presents a case of a broken glass foreign body with rectal injuries caused by self-removal attempts. Standard endoscopic assessment may not be feasible due to the tight impaction and sharp nature of the object. The less invasive transanal approach was selected, which ultimately proved successful for removal.

Significant rectal injury is infrequently caused by retained RFBs. The initial assessment of rectal injury is vital to rule out associated life-threatening complications like perforation or peritonitis, which are associated with a higher mortality risk. A comprehensive evaluation, as highlighted in a recent review by Clark and Maine (20), is essential not only to exclude these acute threats but also to precisely distinguish between intra- and extraperitoneal injury and to grade its severity using the AAST classification. This characterization is crucial as it directly dictates subsequent management decisions (20). Currently, for the management of nondestructive extraperitoneal penetrating rectal injuries specifically, standardized, evidence-based frameworks do exist. The EAST, for instance, has published practice management guidelines for such injuries (9), recommending proximal diversion and avoidance of both routine presacral drains and distal rectal washout. The appropriate intervention is largely determined by the injury grade. For simple, relatively small lacerations close to the anus, Clark and Maine suggest that primary closure be considered (20). For partial-thickness rectal injuries following foreign body insertion, which correspond to grade I according to the AAST classification, nonoperative treatment is recommended by Schellenberg et al. (21). Also, minor lacerations can often heal spontaneously without specific intervention. For instance, Lazzari et al. (22) reported a case similar to the current report, in which a base-up, broken glass cup was safely removed transanally, with only gelatin sponge used for hemostasis of minor mucosal injury.

However, when possible, primary wound closure appears beneficial in the management of less severe extraperitoneal rectal injuries (23). Clark et al. advised that primary repair can be considered for simple, small lacerations close to the anal verge, and a transanal approach may be feasible for nondestructive rectal injuries (20). In this approach, the use of a Lone Star retractor or similar device can evert the mucosa, improving exposure and allowing the surgeon to work more proximally. Turell (24) was the first to describe a detailed technique for managing superficial rectal lacerations. He achieved hemostasis under proctoscopy using a special electrocautery forceps to grasp and coagulate the bleeding wounds. Levine et al. (25) found that primary repair without diversion may be feasible for selected patients with extraperitoneal rectal injury. Hahn et al. (23) successfully performed transanal endoscopic repair of a bleeding rectal laceration with hemoclips. Others have reported transanal endoscopic repair of rectal perforation with endoclipping (26, 27). Additionally, Cheong et al. (28) recommend debridement of non-viable tissue and lavage in cases associated with perineal/anal trauma to prevent sepsis.

In the present case, we decided to keep the rectal wounds open to heal secondarily given the risk of entrapping any potential minute glass fragments, instead of primary closure. The wounds were debrided and then fashioned into a V-shape to ensure sufficient drainage and minimize the pooling of enteric fluid at the wound site. Moreover, the congested and redundant mucosal folds near the wounds were selectively ligated with non-absorbable sutures, aiming to induce organized necrosis and sloughing, thereby removing the tissue that hinders healing. This maneuver is based on the principle of controlled devascularization, a mechanism that is similarly employed in transanal hemorrhoidal dearterialization, where suture ligation of the superior rectal artery branches induces ischemic shrinkage of hemorrhoidal tissue (29, 30). The ligation sites were adequately proximal to the dentate line (more than two centimeters above), resulting in minimal postoperative pain and discomfort. Close postoperative surveillance is needed to detect and manage late complications such as hemorrhage. For instance, Klein et al. (8) presented a similar shattered glass bottle case requiring operative retrieval, which was complicated by severe postoperative hemorrhage. Colonoscopic clipping of a bleeding vessel at a prior laceration site successfully avoided reoperation.

In addition to the technical challenges, such cases also underscore the significance of considering broader patient factors. Patients presenting with RFBs may have underlying psychiatric conditions or intellectual disabilities, which include depression, anxiety, attention-deficit/hyperactivity disorder, and psychosis (3, 7). These issues can contribute to the self-insertion behavior, lead to delayed presentation due to social stigma, and increase the risk of recurrence. As highlighted in the literature (3), a comprehensive psychiatric evaluation and subsequent management are absolutely necessary for all such patients to address root causes and prevent future episodes. Furthermore, vigilance regarding infectious diseases is warranted, particularly in cases associated with high-risk behaviors. As outlined by Lian et al. (31), preoperative screening for blood-borne pathogens (e.g., HBV, HCV, HIV, syphilis) should be performed routinely. This practice is essential not only for comprehensive patient care but also for the safety of healthcare professionals.

While the principles of managing rectal injuries are well established in the existing literature (9, 20), this case highlights the unique challenges posed by a broken glass foreign body, including the fragile nature of the object, the potential for multiple lacerations upon breakage, and the risk of retained small glass fragments. This report calls attention to these specific considerations and demonstrates that wound debridement and leaving wounds open may be a reasonable strategy. Given the inherent limitations of a single case report, definitive conclusions regarding generalizability are unwarranted. Since every injury is unique, the treatment must be individualized according to the specific injury characteristics and patient context.

## Conclusion

Broken glass rectal foreign bodies with associated rectal injuries pose a significant surgical challenge. A systematic approach involving precise diagnosis, cautious intervention, and attentive postoperative care is critical. As demonstrated in the present case, the management strategy combining transanal extraction, wound debridement, and mucosal ligation provides a possibility for curing with a lower risk of complications. However, in cases where severe complications such as perforation are suspected, prompt surgical exploration via laparoscopy or laparotomy remains imperative.

## Data Availability

The original contributions presented in the study are included in the article/Supplementary Material, further inquiries can be directed to the corresponding author.
